# Recovery of Small DNA Fragments from Serum Using Compaction Precipitation

**DOI:** 10.1371/journal.pone.0051863

**Published:** 2012-12-17

**Authors:** Binh V. Vu, Kim L. Anthony, Ulrich Strych, Richard C. Willson

**Affiliations:** 1 Department of Chemical and Biomolecular Engineering, University of Houston, Houston, Texas, United States of America; 2 Department of Biology and Biochemistry, University of Houston, Houston, Texas, United States of America; Barts & The London School of Medicine and Dentistry, Queen Mary University of London, United Kingdom

## Abstract

**Background:**

While most nucleic acids are intracellular, trace amounts of deoxyribonucleic acid (DNA) and ribonucleic acid (RNA), including micro RNAs, can also be found in peripheral blood. Many studies have suggested the potential utility of these circulating nucleic acids in prenatal diagnosis, early cancer detection, and the diagnosis of infectious diseases. However, DNA circulating in blood is usually present at very low concentrations (ng/ml), and is in the form of relatively small fragments (<1,000 bp), making its isolation challenging.

**Methods:**

Here we report an improved method for the isolation of small DNA fragments from serum using selective precipitation by quaternary ammonium compaction agents. A 151 bp fragment of double-stranded DNA from the *Escherichia coli* bacteriophage lambda served as the model DNA in our experiments. DNA was serially diluted in serum until undetectable by conventional polymerase chain reaction (PCR), before being enriched by compaction precipitation.

**Results:**

Starting with concentrations two to three orders of magnitude lower than the PCR-detectable level (0.01 ng/ml), we were able to enrich the DNA to a detectable level using a novel compaction precipitation protocol. The isolated DNA product after compaction precipitation was largely free of serum contaminants and was suitable for downstream applications.

**Conclusions:**

Using compaction precipitation, we were able to isolate and concentrate small DNA from serum, and increase the sensitivity of detection by more than four orders of magnitude. We were able to recover and detect very low levels (0.01 ng/ml) of a small DNA fragment in serum. In addition to being very sensitive, the method is fast, simple, inexpensive, and avoids the use of toxic chemicals.

## Introduction

It is well known that trace amounts of cell-free, circulating deoxyribonucleic acid (DNA), ribonucleic acid (RNA), and micro RNA are present in human blood [1]. The prospect of obtaining prognostic or diagnostic information from these nucleic acids is highly attractive, and previous studies have already established that, for instance, the circulating concentrations of specific nucleotide sequences are significantly higher in prostate, breast and lung cancer patients [2], [3], [4]. There also is extensive work on the value of circulating, trace fetal DNA in the mother’s blood, both as a predictive and as a diagnostic marker [5], [6]. Current methods of isolating cell-free circulating DNA and RNA suffer from low sensitivity and often use toxic chemicals such as phenol and chloroform [7], [8], [9], [10], [11]. Here we report a new, highly-sensitive, simple and inexpensive method to selectively isolate and concentrate small DNA from serum using compaction agents.

Compaction agents are small, cationic molecules which interact selectively with the phosphate backbone and groove of nucleic acid molecules. This interaction can change the conformation of nucleic acids into a compact form which can precipitate out of solution [12]. In the past, compaction agents have been used to selectively precipitate DNA from complex mixtures [13], [14], [15], [16], [17].

## Materials and Methods

### Trace DNA Analyte

To simulate small circulating nucleic acids in blood, we spiked a 151 bp lambda genomic polymerase chain reaction (PCR) amplicon at different concentrations into human serum (Gulf Coast Regional Blood Center, Houston, TX). The fragment was chosen so that the necessary primers would have no significant homology to any human sequences, and was prepared by PCR amplification using total lambda genomic DNA (Affymetrix, Cleveland, OH) as the template. The primers used were: F1, 5′-GGCTTCGGTCCCTTCTGT-3′, and R1, 5′-CTCTTCCAGAAACGACTCCAG-3′ (IDT, Coralville, IA).

In addition, beta-globin-encoding DNA, normally found at low levels in human plasma [18], was used to test the effectiveness of various methods of isolating circulating DNA. For its amplification we used the forward primer 5′-ACACAACTGTGTTCACTAGC-3′ and the reverse primer 5′-CAACTTCATCCACGTTCACC-3′ (IDT, Coralville, IA) [18].

**Figure 1:**
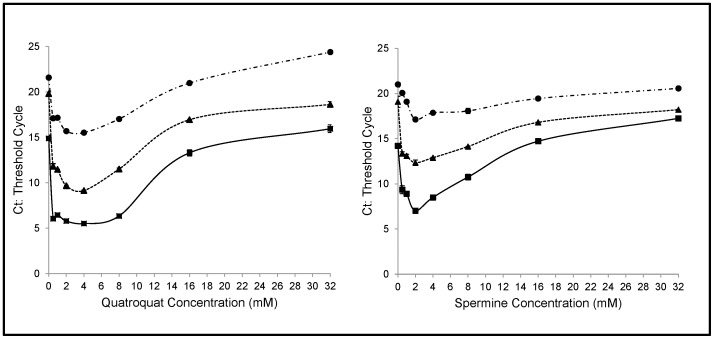
Effect of compaction agent concentration on DNA precipitation. Three sets of human serum samples containing 10, 1, and 0.1 ng/ml of lambda DNA were heat treated and compaction precipitated with various concentrations of spermine and quatroquat.

### Compaction Precipitation

Quatroquat (QQ SBSTM 8915, Sachem, Austin, TX) and spermine-HCl (S1141, Sigma, St. Louis, MO) were used as compaction agents. Various protocols were tested to determine the most effective and efficient method as discussed below. In the final recommended protocol, human serum samples (1 ml) are diluted with nuclease-free water (1.5 ml, OmniPur, EMD Chemicals, Gibbstown, NJ) and heated at 95°C for 15 min to non-specifically precipitate serum proteins. The sample is then vortexed 5 sec, then centrifuged at 16,000×g for 15 min to remove the protein precipitate. The supernatant is recovered and compaction agent is added to a concentration of 2.5 mM. After 15 min incubation on an end-to-end rotator, the samples are again centrifuged at 16,000×g for 15 min. The supernatant is discarded and the DNA pellet is washed with 100 µl Wash Buffer (50% isopropanol, 300 mM NaCl, 10 mM MgCl_2_) and 100 µl 75% ethanol, with centrifugation at 16,000×g for 10 min after each wash. The pellet is then dried in a SpeedVac, before finally being dissolved in 20 µl 10 mM Tris-HCl, pH 7.4.

### Real-time PCR Detection

Real-time PCR for the detection and measurement of DNA was conducted using the Brilliant II SYBR Green® QPCR reagent (Agilent, Santa Clara, CA) in an Mx3000P QPCR system (Agilent). After compaction precipitation, the samples were subjected to real-time PCR without further purification. The original lambda DNA served as a positive control. Primers were the same primers used in preparing the template. All PCRs were set up with 1 µl of sample in 25 µl total volume. PCR conditions were: 95°C for 10 min followed by 35 cycles of 45 s denaturation at 95°C, 1 min annealing at 55°C, followed by 30 s extension at 72°C.

## Results and Discussion

We first investigated the effect of varying the concentration of the compaction agent concentration. Ten ng/ml of a 151 bp lambda model DNA amplicon was serially diluted in serum. The samples were diluted with 1.5 volumes of water and heated to 95°C for 15 min. Denatured aggregated proteins were then removed by centrifugation. The supernatants after centrifugation were precipitated with compaction agents at concentrations between 0 and 32 mM. After analysis of the spiked samples in the various reactions by real-time PCR, we identified an optimal concentration range for each of our compaction agents of 1–5 mM. As shown in Figure 1.A, higher concentrations of spermine noticeably reduced the sensitivity of the PCR. The results for quatroquat showed the same effect (Figure 1.B). It is known that at low and optimal concentration compaction agents interact with the negatively charged DNA backbone to form intermolecular aggregates [14].

Variation of the incubation time of the sample with the compaction agent showed that it had no significant effect on the sensitivity of the subsequent PCR. Serum samples were spiked with 10 ng/ml target DNA, diluted with 1.5 volumes of water, heat-treated as described above, and then divided into two sets, one for each compaction agent. Each set was then subdivided into seven reactions which were treated with 2 mM compaction agent for incubation times ranging from 1 min to 15 hr. Analysis by real-time PCR showed no benefit of prolonged incubation times. In fact, an incubation time of 1 min gave virtually the same level of sensitivity (Ct = 6.68 for spermine, and 6.13 for quatroquat) as a 15 hr incubation (Ct = 6.69 for spermine, and 6.35 for quatroquat) for both compaction agents. We believe that the brief incubation time is a significant advantage of the method; the protocol is simple and can be completed in less than 1.5 hours.

To examine the need for the heat treatment in the protocol, serum samples were aliquoted into two treatment sets: heat treated and not heat treated. Without heat treatment samples subjected to one round of compaction precipitation were detected 3 to 5 cycles later than samples with heat treatment (Table 1). The results showed that heat treatment improved the sensitivity of the real-time PCR. We attribute these results to the removal of inhibitory serum components such as immunoglobulin G and lactoferrin [19], [20] from the PCR by heat. Our real-time PCR results showed that the heat treatment effectively removed these inhibitors, improving the limit of detection by at least an order of magnitude.

**Table 1:** Detection of small DNA in serum after compaction precipitation.

		DNA concentration			
Treatments*		0.01 ng/ml	0.1 ng/ml	1 ng/ml	10 ng/ml
No Heat	No additives	ND	ND	ND	ND
	Sp	21.5±0.4	18±1	16.5±0.7	14±1
	QQ	22.14±0.01	18±1	16.9±0.3	13.5±0.1
	SP (twice)	18.9±0.1	16.0±0.2	12.8±0.5	7.73±0.06
	QQ (twice)	18.61±0.08	16.0±0.1	11.8±0.1	8.56±0.01
Heat	Heat only	ND	ND	33.3±0.2	29.9±0.1
	SP	18.7±0.1	15.7±0.6	11.6±0.2	7.8±0.6
	QQ	18.6±0.1	14.6±0.2	10.37±0.07	6.5±0.2
	SP (twice)	19.5±0.3	16.1±0.1	12.24±0.08	8.4±0.2
	QQ (twice)	17.92±0.05	14.0±0.3	10.1±0.2	6.5±0.1

* Unless otherwise noted (‘twice’) all samples went through one round of the compaction precipitation protocol. Heat: 10 min 95°C. SP: spermine (2 mM), QQ: quatroquat (2 mM), ND: Not detectable.

In optimizing the procedure, we tested a number of variations of the main protocol. As outlined, the original protocol involved heat treatment followed by one round of compaction precipitation. The results were significantly better than heat treatment alone (Table 1). With heat treatment, two rounds of compaction precipitation with either spermine or quatroquat did not improve the outcome (Table 1). Without heat treatment, two rounds of compaction precipitation gave virtually the same results as heat treatment with one round of compaction precipitation. Thus, when heating is not desirable, a second round of compaction precipitation can be substituted.

The possible effect of inter-specimen variation on the compaction precipitation method was examined using 20 distinct serum samples. The samples were divided into four sets, one for each spiked DNA concentration, ranging from 0.01–10 ng/ml. The samples were subjected to purification by compaction precipitation with spermine as described above, followed by real-time PCR. The real-time PCR analysis after compaction precipitation showed no significant variation in DNA recovery among the donors at any of the four DNA concentrations. The average (±SD) Ct values were 6.6±0.2, 10.3±0.7, 15.8±0.9, and 17.6±0.4 for spiked DNA concentrations of 10, 1, 0.1, and 0.01 ng/ml, respectively. Similar results were obtained with quatroquat, the average (±SD) Ct values were 6.6±0.3 for 10 ng/ml, 10.3±0.9 for 1 ng/ml, 14±1 for 0.1 ng/ml, and 17.1±0.5 for 0.01 ng/ml. These results demonstrated the high reproducibility (average coefficient of variation = 5.4%) of the compaction precipitation method across donors.

Compaction precipitation also was compared with the well-developed commercial QIAamp Circulating Nucleic Acid Kit. Pooled serum was spiked with DNA in various concentrations and subjected to DNA purification with compaction precipitation and QIAamp Circulating Nucleic Acid Kit. The results showed that the QIAamp kit was less than 1 cycle more sensitive than our method at 10 ng/ml spiked DNA, and was 2–3 cycles more sensitive at lower concentrations. In an effort to demonstrate the general applicability of the method, the naturally present beta-globin gene was also amplified from blood samples purified by various means. Using the QIAamp Kit, the beta-globin gene was detected at Ct values of 27.2±0.2, with spermine it was detected at Ct values of 28.1±0.4, and with quatroquat at Ct values of 28.0±0.2. Thus, the QIAamp kit was only about one cycle more effective than our compaction precipitation method.

These results were encouraging, since our method is new and not fully optimized. The QIAamp kit employs a large amount of carrier RNA (greater than 1 µg/ml) to enhance recovery, though exogenous RNA would be undesirable in some downstream applications. The addition of carrier RNA to our method did not improve the recovery yield. The cost per sample for the QIAamp kit is hundreds of times higher than compaction precipitation, making our method an attractive choice for high throughput application.

In summary, compaction precipitation proved to be a highly effective method for the isolation and concentration of small DNA fragments from serum. In our final protocol, the combination of heat treatment with one round of compaction precipitation using either spermine or quatroquat improved the sensitivity, especially in samples with initial DNA concentrations between 1–10 ng/ml, the typical concentration range for small DNAs in human blood [21]. Compaction precipitation improved detectability for that concentration range by at least 23 cycles, which is 2^23^ or 6 to 7 orders of magnitude better than heat treatment alone (Table 1). The overall sensitivity of the PCR was also increased by more than five orders of magnitude; specifically, we were able to recover and detect 10 pg/ml of a small DNA fragment in serum. The yield of this method is not subject to significant inter-specimen variation and its initial performance is comparable to that of a widely-used commercial product, at a fraction of the cost. Furthermore, the method is fast, simple, applicable to larger or multiple samples, and avoids the use of hazardous chemicals.

## References

[1] Goebel G, Zitt M, Zitt M, Muller HM (2005) Circulating nucleic acids in plasma or serum (CNAPS) as prognostic and predictive markers in patients with solid neoplasias. Disease Markers 21: 105–120. 16276004 10.1155/2005/218759 PMC3851114

[2] Tamkovich SN, Cherepanova AV, Bryzgunova OE, Kolesnikova EV, Permyakova VI, et al (2008) Deoxyribonuclease activity in biological fluids of healthy donors and cancer patients. Bull Exp Biol Med 146: 89–91. 19145360 10.1007/s10517-008-0213-4

[3] Gal S, Fidler C, Lo YM, Taylor M, Han C, et al (2004) Quantitation of circulating DNA in the serum of breast cancer patients by real-time PCR. Br J Cancer 90: 1211–1215. 15026803 10.1038/sj.bjc.6601609 PMC2409649

[4] Sozzi G, Conte D, Leon M, Ciricione R, Roz L, et al (2003) Quantification of free circulating DNA as a diagnostic marker in lung cancer. J Clin Oncol 21: 3902–3908. 14507943 10.1200/JCO.2003.02.006

[5] Hahn S, Zhong XY, Holzgreve W (2001) Quantification of circulating DNA: in the preparation lies the rub. Clin Chem 47: 1577–1578. 11514390

[6] Lo YMD, Chan KCA, Sun H, Chen EZ, Jiang P, et al (2010) Maternal Plasma DNA Sequencing Reveals the Genome-Wide Genetic and Mutational Profile of the Fetus. Science Translational Medicine 2: 61ra91. 10.1126/scitranslmed.3001720 21148127

[7] Kirsch C, Weickmann S, Schmidt B, Fleischhacker M (2008) An improved method for the isolation of free-circulating plasma DNA and cell-free DNA from other body fluids. Circulating Nucleic Acids in Plasma and Serum V 1137: 135–139. 10.1196/annals.1448.035 18837937

[8] Schmidt B, Weickmann S, Witt C, Fleischhacker M (2005) Improved method for isolating cell-free DNA. Clinical Chemistry 51: 1561–1563. 16040863 10.1373/clinchem.2005.051003

[9] Stroun M, Anker P, Lyautey J, Lederrey C, Maurice PA (1987) Isolation and Characterization of DNA from the Plasma of Cancer-Patients. European Journal of Cancer & Clinical Oncology 23: 707–712. 3653190 10.1016/0277-5379(87)90266-5

[10] (2009) QIAamp® Circulating Nucleic Acid Handbook.

[11] (2010) Circulating DNA from Plasma. NucleoSpin® Plasma XS User Manual: Macherey-Nagel.

[12] Gosule LC, Schellman JA (1976) Compact Form of DNA Induced by Spermidine. Nature 259: 333–335. 1250371 10.1038/259333a0

[13] Anez-Lingerfelt M, Fox GE, Willson RC (2009) Reduction of DNA contamination in RNA samples for reverse transcription-polymerase chain reaction using selective precipitation by compaction agents. Analytical Biochemistry 384: 79–85. 18831957 10.1016/j.ab.2008.09.009 PMC5476456

[14] Hoopes BC, Mcclure WR (1981) Studies on the Selectivity of DNA Precipitation by Spermine. Nucleic Acids Research 9: 5493–5504. 7029471 10.1093/nar/9.20.5493 PMC327535

[15] Mourich DV, Munks MW, Murphy JC, Willson RC, Hill AB (2003) Spermine compaction is an efficient and economical method of producing vaccination-grade DNA. Journal of Immunological Methods 274: 257–264. 12609551 10.1016/s0022-1759(02)00516-1

[16] Murphy JC, Wibbenmeyer JA, Fox GE, Willson RC (1999) Purification of plasmid DNA using selective precipitation by compaction agents - A scaleable method for the liquid-phase separation of plasmid DNA from RNA. Nature Biotechnology 17: 822–823. 10.1038/11777 10429254

[17] Razin S, Rozansky R (1959) Mechanism of the antibacterial action of spermine. Archives of Biochemistry and Biophysics 81: 36–54. 13637964 10.1016/0003-9861(59)90173-0

[18] Jung M, Klotzek S, Lewandowski M, Fleischhacker M, Jung K (2003) Changes in concentration of DNA in serum and plasma during storage of blood samples. Clinical Chemistry 49: 1028–1029. 12766024 10.1373/49.6.1028

[19] Al-Soud WA, Jonsson LJ, Radstrom P (2000) Identification and characterization of immunoglobulin G in blood as a major inhibitor of diagnostic PCR. Journal of Clinical Microbiology 38: 345–350. 10618113 10.1128/jcm.38.1.345-350.2000 PMC88721

[20] Al-Soud WA, Radstrom P (2001) Purification and characterization of PCR-inhibitory components in blood cells. Journal of Clinical Microbiology 39: 485–493. 11158094 10.1128/JCM.39.2.485-493.2001 PMC87763

[21] Leon SA, Shapiro B, Sklaroff DM, Yaros MJ (1977) Free DNA in the serum of cancer patients and the effect of therapy. Cancer Res 37: 646–650. 837366

